# Lack of Galectin-3 Drives Response to *Paracoccidioides brasiliensis* toward a Th2-Biased Immunity

**DOI:** 10.1371/journal.pone.0004519

**Published:** 2009-02-20

**Authors:** Luciana Pereira Ruas, Emerson Soares Bernardes, Marise Lopes Fermino, Leandro Licursi de Oliveira, Daniel K. Hsu, Fu-Tong Liu, Roger Chammas, Maria-Cristina Roque-Barreira

**Affiliations:** 1 Departamento de Biologia Celular e Molecular e Bioagentes Patogênicos, Faculdade de Medicina de Ribeirão Preto, Universidade de São Paulo, Ribeirão Preto, Brasil; 2 Departamento de Biologia Geral, Universidade Federal de Viçosa, Viçosa, Brasil; 3 Department of Dermatology, School of Medicine, University of California Davis, Sacramento, California, United States of America; 4 Laboratório de Oncologia Experimental, Faculdade de Medicina, Universidade de São Paulo, São Paulo, Brasil; Albert Einstein College of Medicine, United States of America

## Abstract

There is recent evidence that galectin-3 participates in immunity to infections, mostly by tuning cytokine production. We studied the balance of Th1/Th2 responses to *P. brasiliensis* experimental infection in the absence of galectin-3. The intermediate resistance to the fungal infection presented by C57BL/6 mice, associated with the development of a mixed type of immunity, was replaced with susceptibility to infection and a Th2-polarized immune response, in galectin-3-deficient (gal3^−/−^) mice. Such a response was associated with defective inflammatory and delayed type hypersensitivity (DTH) reactions, high IL-4 and GATA-3 expression and low nitric oxide production in the organs of infected animals. Gal3^−/−^ macrophages exhibited higher TLR2 transcript levels and IL-10 production compared to wild-type macrophages after stimulation with *P. brasiliensis* antigens. We hypothesize that, during an *in vivo P. brasiliensis* infection, galectin-3 exerts its tuning role on immunity by interfering with the generation of regulatory macrophages, thus hindering the consequent Th2-polarized type of response.

## Introduction

*Paracoccidioides brasiliensis*, a thermally dimorphic fungus, is the etiological agent of human paracoccidioidomycosis, one of the most frequent systemic mycosis in Central and South America [1], [2]. The main host defense against *P. brasiliensis* is the cell-mediated immune response [3]–[5]. Macrophage activation and granuloma formation characterize the inflammatory response induced by the fungus, and protect the host against parasite dissemination [6]. Macrophages and lymphocytes are generally considered the major effector cells controlling the disease *in vivo* [7], [8] through TNF-α and IFN-γ production [9], [10].

In the past few years, the immunity to several infections has been demonstrated to be influenced by galectin-3, the most studied member of the galectin family. These investigations were performed by comparing the course of experimental infections in mice that were genetically deficient, or not, in galectin-3 [11]–[13]. The studies of infection with the intracellular bacteria *Rhodococcus equi* have demonstrated that galectin-3 may regulate the innate immune response by diminishing IL-1β production by macrophages [13]. On the other hand, during *Toxoplasma gondii* and *Schistosoma mansonii* infection, the absence of galectin-3 drives the development of a heightened Th1-type immune response, suggesting that the lectin exerts a profound effect on the development of the adaptive immune response against pathogens [11], [12]. Together with results obtained from a murine model of asthma [14], the studies provide consistent evidences that gal3^−/−^ mice develop a lower Th2 response but a higher Th1 response compared with gal3^+/+^ mice. In consequence, galectin-3 emerges as a fine regulator of Th1/Th2 balance.

Since protection against *P. brasiliensis* infection depends on adequate inflammation, cellular immune response and cytokine production, and because galectin-3 is important in the regulation of the Th1/Th2 balance, the aim of this study was to analyze the immunological aspects of *P. brasiliensis* infection in galectin-3-deficient (gal3^−/−^) mice. We demonstrate that gal3^−/−^ mice present increased susceptibility to *P. brasiliensis* infection, associated with the inability to mount an adequate inflammatory response, the impairment of DTH response, high serum levels of specific antibodies, and the development of a Th2-polarized immune response. Such a picture is possibly due to the fact that, following contact with *P. brasiliensis* antigens, macrophages from gal3^−/−^ mice have higher TLR2 transcript levels and produce higher levels of the deactivating cytokine IL-10. Our results indicate that galectin-3 exerts a protective and immunoregulatory role in the host response to *P. brasiliensis* infection.

## Materials and Methods

### Experimental Animals

Galectin-3-deficient mice (gal3^−/−^) were generated as previously described and backcrossed to C57BL/6 mice for nine generations [15]. Age-matched wild-type mice C57BL/6 (gal3^+/+^) were used as controls in all the experiments. Mice were housed under approved conditions in the Animal Research Facilities of Faculdade de Medicina de Ribeirão Preto - USP. All of the animals used in the experiments were male, at 6 to 8 week-old. The Ethics Committee on Animal Research of the University of São Paulo approved all the procedures performed in the studies described here.

### Parasite, Mice Infection, and Mortality

The *P. brasiliensis* isolate 18 (Pb18), which is highly virulent, was used throughout this study. Yeasts cells were maintained by weekly subcultivation in a semisolid culture medium [16] at 36°C and were used on day 7 of culturing. They were harvested and washed three times with phosphate-buffered saline (PBS), pH 7.2. Cell viability was determined as previously described [17]. Mice were infected intraperitoneally (i.p.) with 5×10^6^ viable yeast cells in 500 µL PBS and control animals received PBS only. Animal death was registered daily until 120 days after infection. For the intratracheal (i.t.) infection assay, mice were anesthetized by intraperitoneal injection with 2.5% of tribromoethanol and infected with 5×10^6^ yeast cells suspended in PBS.

### Antigen Preparation

*P. brasiliensis*-antigen (PbAg) was obtained as previously described [16]. Briefly, Pb18 yeast cells were harvested, washed with PBS, disrupted by ultrasonic treatment (5 cycles of 30 seconds), and centrifuged for 10 min at 2000×*g*. The supernatants were collected and the protein concentration was determined by the Bicinchoninic acid assay (BCA) (Pierce, Rockford, IL, USA), according to the manufacturer's protocol.

### Tissue Processing

Groups of gal3^+/+^ and gal3^−/−^ mice were euthanized 4 weeks postinfection. The lungs obtained from different groups of mice were fixed in 10% buffered formalin for 24 hours and processed routinely for paraffin embedding and sectioning. Five-micrometer sections were stained with hematoxylin and eosin (H&E) for analysis of granulomatous lesions and intensity of inflammatory infiltrates.

### Immunohistochemistry for Detection of Galectin-3 in Tissues of Mice

To detect galectin-3 expression by immunohistochemistry, deparaffinized sections were incubated in 2% unlabeled goat serum diluted in 1% Bovine Serum Albumin (BSA) for 1 hour to reduce nonspecific binding. The slides were then incubated with rat anti-mouse galectin-3 mAb (M3/38) [18] diluted in PBS containing 1% BSA for one hour. The secondary antibodies labeled with peroxidase were goat anti-rat IgG antibodies (Sigma Chemical Co., St. Louis, MO, USA). The reaction was visualized by incubating the section with 3,3′-diaminobenzidine tetrahydrochloride (Pierce, Rockford, IL, USA) in PBS+H_2_O_2_ for 30 min. In the control slides, normal rat IgG replaced the primary antibody. The slides were viewed by light microscopy using an Olympus BX50 microscope (Olympus Instruments, Melville, NY, USA) equipped with a digital camera Nikon DMX 1200 (Nikon, Melville, NY, USA). All steps were performed at room temperature.

### Assay for Organ Colony-Forming Units

To assay the dissemination of the fungus to the lung and liver, the animals were euthanized after 30 days of both i.p and i.t. infection and the organs were removed, weighed, homogeneized in sterile PBS (pH 7.2) and serially diluted. Aliquots of 100 µL were dispensed into Petri dishes, in duplicates, containing brain-heart infusion agar (Difco Laboratories, Detroit, MI, USA) supplemented with 4% (v/v) of fetal bovine serum. Plates were incubated at 37°C, and colonies were counted 7 and 14 days later. Results are expressed as the number of colony-forming units (CFU)±SD per gram of tissue.

### Quantification of P. brasiliensis by real time PCR

Molecular quantification of *P. brasiliensis* was performed as previously described [19]. Lung samples were frozen in liquid nitrogen for 30 s and pulverized. The total DNA was extracted and precipitated using the DNeasy Tissue Kit (Qiagen, Valencia, CA, USA), according to the manufacturer's protocol. PCR amplification and analysis were achieved using an ABI Prism 7500 sequence detector (Applied Biosystems, Foster City, CA, USA). Reactions were performed with TaqMan Universal PCR Master Mix (Applied Biosystems) in 20 µL solutions containing 50 ng of template DNA, 5 pmol of each primer, and probe and 10 µL of TaqMan Master Mix. Each sample was tested in duplicate and all quantifications were normalized to an endogenous control (β-actin). The primers and probes used for PCR amplification targeting the Gp43 gene [20] for *P. brasiliensis* and β-actin gene for mouse were designed using the Primer Express® software v2.0 (Applied Biosystems). The sequences for primers and probe for β-actin gene are: forward - 5′- AGCTGCGTTTTACACCCTTT -3′; reverse - 5′- AAGCCATGCCAATGTTGTCT -3′ and probe: -5-FAM-TGACAAAACCTAACTTGCGCAGAAAAA-Tamra-3′. The primers and probe for Gp43 gene are: forward - 5′- FAM-GATTGATGAAGCTGCGGTTGA-Tamra-3′ and reverse 5′- CATACAGATCTCCGACGCTGC -3′.

### Delayed-Type Hypersensitivity (DTH) Assay

DTH responses against *P. brasiliensis* antigens were evaluated 90 days after infection by the footpad test, as previously described [21]. The left hind footpad was injected subcutaneously with 25 µL PbAg. Swelling was evaluated by measuring the footpad thickness just before and 24 h after injection of the antigen, using a dial caliper (Mitutoyo, Tokyo, Japan). The increase in thickness was calculated. The same procedure was performed in non-infected control mice.

### Quantification of P. brasiliensis-specific IgG1 and IgG2b

Specific anti-*P. brasiliensis* IgG1 and IgG2b antibodies in the serum samples were detected by enzyme-linked immunosorbent assay (ELISA). Microtiter plates (Nunc, Naperville, IL, USA) were coated with PbAg (50 µL per well) at a concentration of 20 µg/mL in 50 mM sodium carbonate buffer, pH 9.6, overnight, at 4°C. The plates were then washed three times with PBS containing 0.05% Tween 20 (PBS-T) (pH 7.4). Non-specific binding sites were blocked with PBS-T containing 1% BSA (blocking buffer) for 1 h, at 37°C. Serum samples were added to duplicate wells at a 1/90 dilution in blocking buffer. Plates were then incubated at 37°C for 1 h, washed four times, and incubated with peroxidase-conjugated goat anti-mouse IgG1 or IgG2b antibody (Santa Cruz Biotechnology, Santa Cruz, CA, USA) in a 1/5000 dilution in blocking buffer for 1 h, at 37°C. After washing with PBS-T, reactions were developed with the TMB (3,3′,5,5′-tetramethylbenzidine) Substrate Kit according to the manufacturer's instructions (Pierce Chemical Co., Rockford, IL, USA). The reaction was stopped 15 min later by addition of 25 µL of 1 M sulfuric acid to each well. The absorbance at 450 nm was read in a Microplate Scanning Spectrophotometer (PowerWave X, Bio-Tek Instruments, Inc., Winooski, VT, USA). As a reaction control, antigen coated plates were incubated with serum of non-infected mice. An additional control was provided by BSA coated wells incubated with serum of infected mice.

### Cytokine Detection in Organ and Peritoneal Macrophage Supernatants

Cytokine concentrations in the tissues were quantified with a commercially available kit, according to the manufacturer's instructions (OptEIA set; Pharmingen, San Diego, CA, USA). To evaluate secretion of the IFN-γ and IL-4 cytokines in the lungs, the organs were weighed and homogenized in 1 mL of complete inhibitor cocktail buffer (Boehringer, Mannheim, Germany) using a tissue homogenizer. The samples were centrifuged at 5000×*g* for 10 minutes and assayed for cytokine production. For cytokine quantification in the macrophage supernatants, cells were stimulated *in vitro* for 48 hours and IL-10 content was then determined. The concentrations were determined by comparison with standard curves constructed with known amounts of the respective mouse recombinant cytokines. The assay sensitivity limits were 7 pg/mL for IL-4, and 30 pg/mL for IFN-γ and IL-10.

### Real Time quantitative PCR analysis

Total RNA was isolated from 2×10^6^ cells using TRIzol reagent (Invitrogen Life Technologies, Carlsbad, CA, USA), following the manufacturer's intructions. cDNA synthesis was performed in a final volume of 20 µL, using ImProm-II Reverse Transcriptase (Promega Corporation, Madison, WI, USA). The reaction mixture contained 4 µg of total RNA, 20 pmol of oligo dT primer (Invitrogen Life Technologies, Carlsbad, CA, USA), 40 U of RNAsin, 1 M of dNTP mix, and 1 U of reverse transcriptase buffer. cDNA was treated with 10 µg of RNase (Gibco, Carlsbad, CA, USA). It was then immediately used or stored at −20°C. PCR amplification and analysis were achieved using an ABI Prism 7500 sequence detector (Applied Biosystems). All the reactions were performed with SYBR Green Master Mix (Applied Biosystems) using a 25 µL volume in each reaction, which contained 2 µL of template cDNA, 5 pmol of each primer, and 12.5 µL of SYBR Green. The primers used for PCR amplification were as follows: for IL-4, 5′- GTCTCTCGTCACTGACGGCA- 3′ (forward) and 5′- CGTGGATATGGCTCCTGGTAC -3′ (reverse); for GATA-3, 5′- AAGAAAGGCATGAAGGACGC -3′ (forward) and 5′- GTGTGCCCATTTGGACATCA -3′ (reverse); for arginase I, 5′- CAAAAGGACAGCCTCGAGGAG- 3′ (forward) and 5′- CCCGTGGTCTCTCACGTCAT -3′ (reverse); for TLR2, 5′- CTCTGACCCGCCCTTTAAGC - 3′ (forward) and 5′- TTTTGTGGCTCTTTTCGATGG - 3′ (reverse); for β-actin, 5′- AGCTGCGTTTTACACCCTTT -3′ (forward) and 5′- AAGCCATGCCAATGTTGTCT -3′ (reverse).

### Mouse Peritoneal Macrophages

Groups of gal3^+/+^ and gal3^−/−^ mice were i.p. injected with 1 mL of sterile 3% sodium thioglycollate (Sigma Chemical Co). After 4 days, mice were euthanized and their cells were recovered by peritoneal lavage using 5 mL of ice-cold Hank's balanced salt solution (HBSS). Cells were immediately stored on ice, washed in HBSS, suspended in RPMI-5% medium, and dispensed in 24-well cell culture plates (2×10^6^ cells/well). After a 4 h incubation at 37°C, nonadherent cells were gently removed, and the adherent cells were incubated in RPMI-5% either in the presence or absence of cells from Pb18 culture (8×10^6^ cells/well) or PbAg (20 µg/mL). They were then cultured at 37°C in a humidified 5% CO_2_ atmosphere. The culture supernatants were collected after 24 and 48 hours for NO determination and IL-10 quantification, as described below.

### Nitrite Content in Organ and in the Supernatant of Mouse Macrophage Culture

NO production was quantified by accumulation of nitrite, using the standard Griess reaction. Briefly, 50 µL of the supernatants were incubated with an equal volume of the Griess reagent (1% sulfanilamide, 0.1% naphthyl ethylenediamine dihydrochloride, 2.5% H_3_PO_4_) for 10 min, at room temperature. The absorbance was measured at 550 nm in the Microplate scanning spectrophotometer. The conversion of absorbance into micromolar concentrations of NO was deduced from a standard curve using a known concentration of NaNO_2_ diluted in RPMI medium.

### Statistical Analysis

The results are expressed as the mean±SD of the indicated number of animals or experiments. Statistical analysis was performed using analysis of variance followed by the parametric Tukey-Kramer test (INSTAT software, GraphPad, San Diego, CA, USA). A *P* value<0.05 was considered statistically significant. The log rank test was used to compare the survival rates between the study groups, and differences were considered statistically significant when *P*<0.05.

## Results

### Galectin-3 is Involved in the Resistance to Infection with *Paracoccidioides brasiliensis*

**Figure 1:**
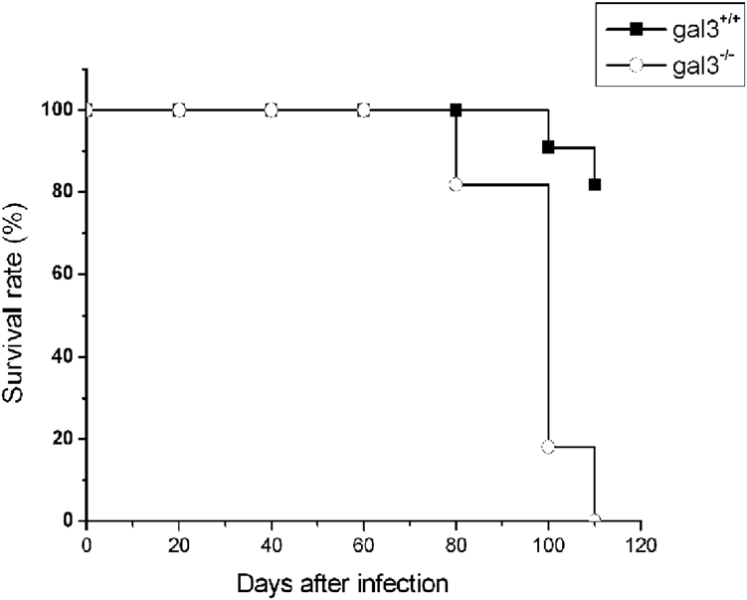
Absence of galectin-3 leads to lower survival in mice infected by *P. brasiliensis*. The survival rate of gal3^+/+^ (black squares) and gal3^−/−^ (open squares) mice after intraperitoneal infection with 5×10^6^ yeasts cells (P<0.0001, compared with infected gal3^+/+^ mice.). Data are representative of two experiments, each performed with eight to ten mice per group.

The effect of galectin-3 in host survival to *P. brasiliensis* infection was studied in mice deficient in the galectin-3 gene. At 80 days post-infection, 20% of the gal3^−/−^ mice died, whereas all wild-type (gal3^+/+^) mice survived until 100 days after infection (Figure 1). By day 110, gal3^−/−^ mice reached 100% mortality while 80% of gal3^+/+^ mice survived in the same period of infection. Because galectin-3 deficiency was associated with higher vulnerability of mice to the infection, we hypothesize that galectin-3 contributes to the host resistance against the fungus.

### Galectin-3 Controls the Pulmonary Inflammatory Reaction and Fungal Burden in the *P. brasiliensis* Infected Mice

**Figure 2:**
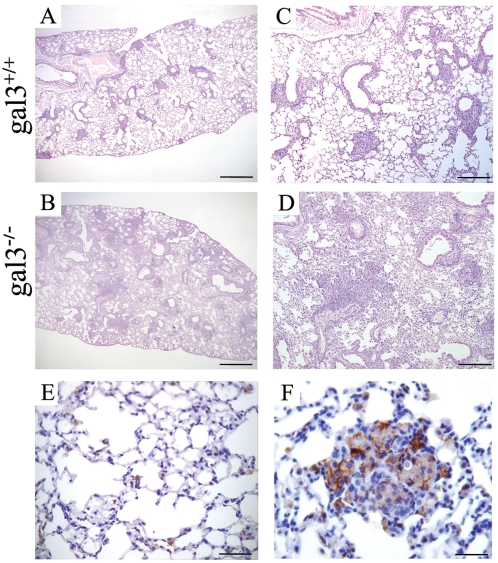
Histopathology and immunohistochemistry staining for galectin-3 in lung sections from *P. brasiliensis*-infected gal3^+/+^ and gal3^−/−^ mice. Mice were intraperitoneally infected with 5×10^6^ yeasts cells and their lungs were analyzed after 30 days. Lungs of gal3^+/+^ mice show well-organized granulomas (A and C). Gal3^−/−^ mice present a diffuse pattern of inflammatory cells, with incipient granuloma formation (B and D). Galectin-3 staining increases after infection with the fungus, mostly detected in the inflammatory cells of the granulomas and surrounding yeast cells (F). Galectin-3 is barely noted in the lungs of non-infected control mice (E). Sections were stained with H&E (A–D) or immunostained for galectin-3 with a hematoxilin counterstain (E–F). Scale bars on panels A and B indicates 1 mm; on C and D 400 µm, and on E and F 100 µm.

Because the formation of a compact granuloma is crucial during *P. brasiliensis* infection [22], we compared the inflammatory response in the lungs of infected gal3^−/−^ and gal3^+/+^ mice. At 30 days post-infection, the lung tissue of gal3^+/+^ mice exhibited compact and individualized granulomatous lesions (Figure 2A and 2C), while the lungs of gal3^−/−^ mice showed a diffuse inflammatory reaction, with incomplete organization of the granuloma. The lesions were confluent and poorly structured, causing a disruption of the pulmonary parenchyma (Figures 2B and 2D). Immunohistochemistry staining revealed an increased amount of galectin-3 in the lungs of gal3^+/+^ infected mice compared with non-infected controls (Figures 2E and 2F). As expected, no staining was observed in the lungs of infected or non-infected gal3^−/−^ mice (not shown).

**Figure 3:**
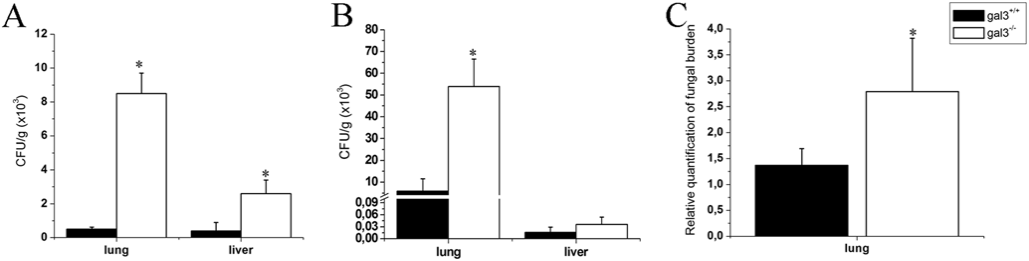
Absence of galectin-3 leads to increased *P. brasiliensis* burden. Colony-forming units (CFU) recovered from lungs and liver (A) of gal3^+/+^ (black bars) and gal3^−/−^ (open bars) mice 30 days after intraperitoneal (A) and intratracheal (B) infection with yeast cells. Relative quantification of *P. brasiliensis* in the lungs of infected mice 30 days after i.p. infection was carried out through real-time PCR (C). The bars represent the mean±SD of CFU obtained from duplicate samples in groups of five animals. * P<0.05, compared with infected gal3^+/+^ mice.

The fungal CFU recovered from the lungs and liver were dramatically higher in gal3^−/−^ mice compared with gal3^+/+^ mice (Figure 3A), an observation that was also confirmed after infection by the intratracheal route and by quantitative real-time PCR specific for *P. brasiliensis* DNA (Figure 3B and C). These results indicate that galectin-3 is involved in the development of an effective inflammatory reaction and in the control of fungal burden.

### Cellular and Humoral Response against *P. brasiliensis* is influenced by Galectin-3

**Figure 4:**
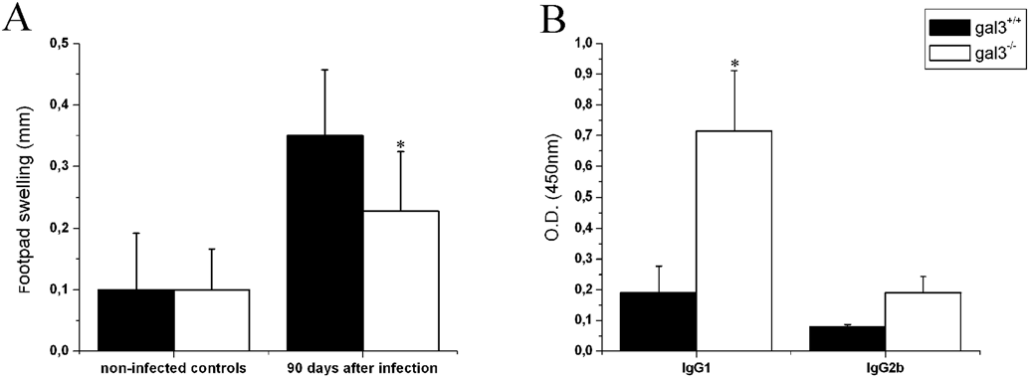
Cellular and humoral responses to *P. brasiliensis* in the absence of galectin-3. DTH reaction (A) and serum level of specific antibodies (B) of gal3^+/+^ (black bars) and gal3^−/−^ (open bars) mice intraperitoneally infected with 5×10^6^ yeasts cells. DTH reaction was assayed in groups of 8–13 mice, 90 days after infection. Each bar represents the mean±SD from a representative experiment of two assays. IgG1 and IgG2b levels were detected by ELISA in the sera of mice 30 days after infection. The bars represent the mean±SD of the O.D. obtained for sera, diluted 30 times, of five animals and are representative of two independent experiments. * P<0.05, compared with infected gal3^+/+^ mice.

Next, we evaluated whether the immune response against the fungus is affected by galectin-3. The DTH response was evaluated 90 days post-infection, by measuring the footpad thickness developed after the local injection of the fungal antigens. The thickness was significantly lower (*P*<0.05) in gal3^−/−^ mice compared with gal3^+/+^ mice, whereas no difference was observed between the groups of non-infected mice (Figure 4A). The determination of the serum levels of antibodies reacting with the fungal antigens showed that by day 30 post-infection, gal3^−/−^ mice had significantly (*P*<0.05) higher levels of specific IgG1 compared to gal3^+/+^ mice (Figure 4B). The former also developed a higher antigen-specific IgG2b response, compared to the latter, but the difference was not statistically significant. No reaction with fungal antigens occurred with the serum of the non-infected mice or following incubation of serum samples of infected mice with BSA coated plates (not shown). Because lower cellular immunity and higher levels of IgG1 antibodies were observed in gal3^−/−^ mice, suggesting that a Th2 response could be mounted, we hypothesize that galectin-3 is required to sustain the immunity responsible for the intermediate susceptibility of C57BL/6 mice to *P. brasiliensis* infection.

### A Th2-polarized Immune Response during *P. brasiliensis* infection is associated with the absence of Galectin-3

**Figure 5:**
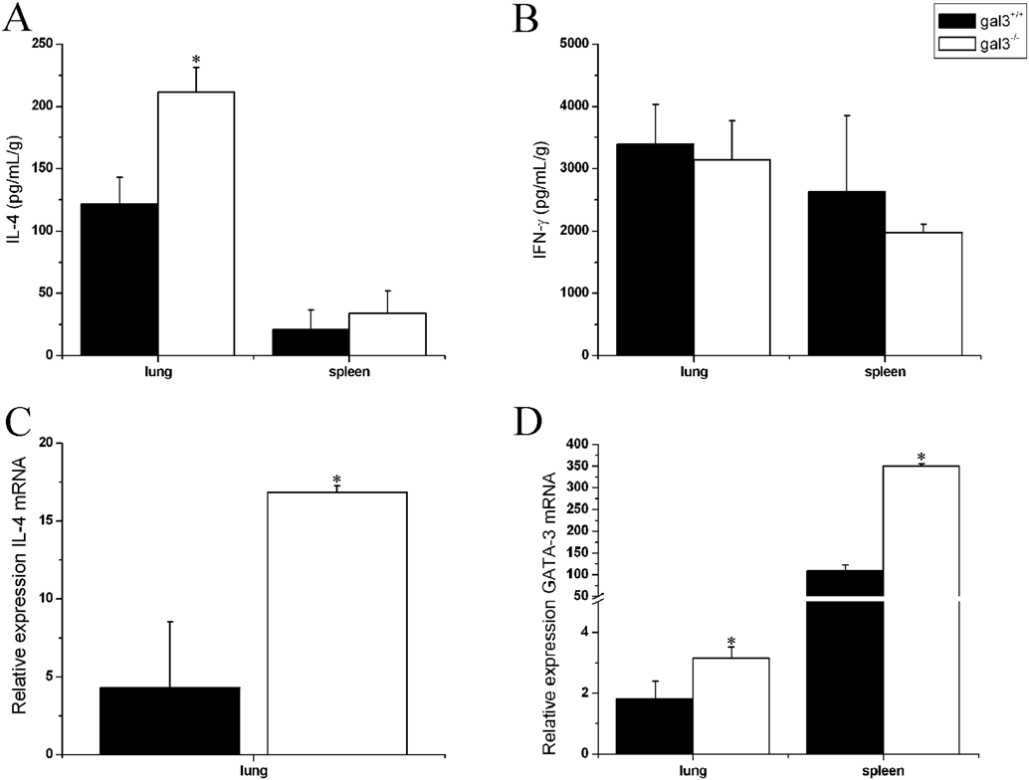
Levels of IL-4 (A and C), IFN-γ (B) and GATA-3 (D) in the organs of *P. brasiliensis*-infected gal3^+/+^ and gal3^−/−^ mice. Mice were intraperitoneally infected with 5×10^6^ yeasts cells and samples of their organs weighed and homogenized. Protein levels of IL-4 and IFN-γ in the lungs and spleen of 30 day-infected mice were assayed by ELISA (A and B). The levels of mRNA relative expression for the IL-4 and GATA-3 in the lungs (30 days after infection) and GATA-3 in the spleen (7 days after infection) of infected mice were determined by real-time PCR, using the β-actin gene as control. The results represent the mean±SD of three to five mice per group, from a representative experiment of three assays. * P<0.05, compared with infected gal3^+/+^ mice.

To better characterize the type of immune response mounted by gal3^−/−^ C57BL/6 mice during *P. brasiliensis* infection, we determined the levels of cytokines in the spleen and lung homogenates of gal3^+/+^ and gal3^−/−^ mice, obtained 30 days after fungal inoculation. We found higher levels of IL-4 and IFN-γ in both groups of infected mice compared with the non-infected ones (data not shown). Higher amounts of IL-4 were detected in the lungs of *P. brasiliensis*-infected gal3^−/−^ mice compared with infected gal3^+/+^ mice (*P*<0.05) (Figure 5A), a result that was supported by the higher relative expression of IL-4 mRNA found in the lungs of infected gal3^−/−^ mice (Figure 5C). IFN-γ levels detected in the spleen and lungs of gal3^+/+^ and gal3^−/−^ mice were not statistically different (Figure 5B). However, we found a higher relative expression of the T cell transcriptional factor GATA-3 in the spleen and lung of infected gal3^−/−^ mice in comparison to gal3^+/+^ mice (Figure 5D). These results suggest that gal3^−/−^ mice develop a Th2-polarized immune response when infected with *P. brasiliensis*, a fact that is consistent with the increased susceptibility of these mice to the fungal disease.

### Galectin-3 is Required for NO Production Induced by Fungal Antigens

**Figure 6:**
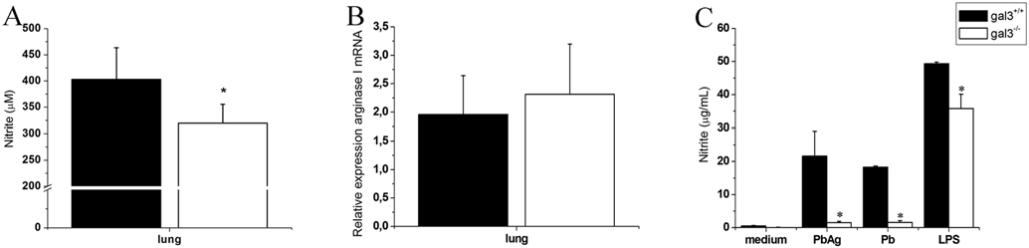
NO production (A) and arginase I levels (B) detected in the lungs of *P. brasiliensis*-infected mice and production of NO *in vitro* by peritoneal macrophages (C). In panel A, nitrite levels were detected by the Griess method in the lungs of mice, after 30 days of infection. The results represent the mean±SD of eight mice per group, from a representative experiment of three separate assays. Panel B shows transcript levels of arginase I mRNA in the lungs of mice, after 30 days of infection, determined by real-time PCR, using the β-actin gene as control. In panel C, nitrite levels were detected in the supernatant of adherent cells harvested from the peritoneal cavity of gal3^+/+^ (black bars) and gal3^−/−^ (open bars) mice treated with thioglycollate. Cell cultures were incubated for 48 hours with medium alone or stimulated with bacterial LPS (1 µg/mL) or *P. brasiliensis* antigens (PbAg, 20 µg/mL) or *P. brasiliensis* yeast (Pb, 8×10^7^ cells/mL). Each bar represents the mean±SD from a representative experiment of two assays. * P<0.05, compared with infected gal3^+/+^ mice.

To better evaluate the effect of galectin-3 in the mechanisms of resistance to *P. brasiliensis* infection, we measured the pulmonary NO levels in the infected mice 30 days after infection. NO levels produced by gal3^−/−^ mice were significantly lower than those produced by gal3^+/+^ mice (Figure 6A). No significant difference was found regarding the arginase I mRNA expression in the lungs of mice from both groups (Figure 6B). To determine whether galectin-3 could directly interfere with this effector mechanism of resistance, we measured the *in vitro* NO production by peritoneal macrophages co-cultured with *P. brasiliensis* or stimulated with fungal antigens. In both conditions, peritoneal macrophages obtained from gal3^+/+^ mice produced higher NO levels (Figure 6C), a response that was reproduced by cells stimulated with LPS (positive control). These results give evidence for an additional mechanism by which galectin-3 contributes to resistance to the fungal infection.

### *P. brasiliensis* Antigens Induce Higher TLR2 Transcript Expression in Gal3^−/−^ Macrophages, Associated with Higher Levels of IL-10

**Figure 7:**
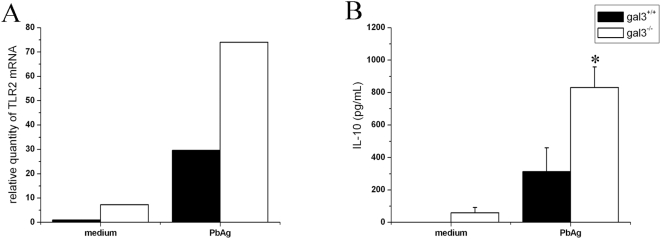
*P. brasiliensis* antigens induce higher TLR2 transcript levels in gal3^−/−^ thioglycollate-elicited macrophages. (A) Real Time quantitative RT PCR for TLR2 using mRNA from unstimulated or PbAg (20 µg/mL)-treated macrophages, 4 hours after stimulation. cDNA contents were normalized on the basis of predetermined levels of β-actin. TLR2 transcript level was higher in PbAg-stimulated gal3^−/−^ macrophages (open bars) compared with gal3^+/+^ macrophages (black bars). (B) Thioglycollate-elicited macrophages from gal3^+/+^ (black bars) and gal3^−/−^ (open bars) mice were incubated with the indicated stimuli for 48 hours; IL-10 was quantified in the cell supernatants by ELISA. Results represent the mean±SD obtained from duplicate wells. Results are representative of two independent experiments that provide similar results. * P<0.05, compared with infected gal3^+/+^ mice.

Because *P. brasiliensis* antigens induced the down regulation of NO production in gal3^−/−^ mice, we investigated whether they were able to interfere with TLR2 expression in macrophages. Previous results from our group had shown that TLR2 expression is altered in galectin-3-deficient macrophages infected with intracellular bacteria *Rhodococcus equi* [13]. Here we found, in non-stimulated macrophages, higher TLR2 mRNA expression in gal3^−/−^ compared to gal3^+/+^ cells (Figure 7A). *P. brasiliensis* antigen increased the expression of TLR2 in both genotypes of macrophages. Remarkably, gal3^−/−^ macrophages stimulated with PbAg contained the transcript level at 2.5 times higher than that detected in gal3^+/+^ macrophages (Figure 7A). These results indicate that galectin-3 regulates TLR2 expression in macrophages, mainly when they are stimulated with fungal antigen. IL-10 measurement in the supernatants of macrophages show that the level produced by gal3^+/+^ macrophages was almost 2.6 times lower compared with gal3^−/−^ cells (Figure 7B). The results determined by PbAg were reproduced by the stimulation with *P. brasiliensis* yeast (not shown). These results suggest that galectin-3, in addition to controlling the TLR2 expression on macrophages stimulated with fungal antigen, regulates the IL-10 production by these cells.

## Discussion

In this work, we describe how *P. brasiliensis* infection can be influenced by the role exerted by galectin-3 on the host immunity. In the absence of galectin-3, the pattern of immune response of C57BL/6 mice to *P. brasiliensis* infection changes. The known intermediate resistance of C57BL/6 mice to *P. brasiliensis* infection [23], associated with a mixed type of immune response, is substituted by clear susceptibility to the fungal infection in gal3^−/−^ C57BL/6 mice, associated with a Th2-polarized immune response.

The greater susceptibility to *P. brasiliensis* infection in the absence of galectin-3 may be attributed to a combination of factors, including impairment of both inflammatory reaction and cellular specific immune response, whose integrity is critical for protection against the fungus [5], [22]. After *P. brasiliensis* infection, the diffuse inflammatory reaction found in the lungs of gal3^−/−^ mice contrasts with the well delineated granulomas observed in wild-type mice, which restrains fungal dissemination, as indicated by a lower fungal burden. A depressed DTH response to fungal antigens observed in gal3^−/−^ infected mice is consistent with the cellular immune response defect repetitively found in paracoccidioidomycosis models developed in susceptible mice [21], [24], [25]. Such models mimic the polar forms of the human disease and are correlated with an elevated production of IgG1 and IgG2b antibodies [24]. Compared with gal3^+/+^ mice, gal3^−/−^ mice have lower DTH responses and produce higher titers of IgG1. This state is compatible with the occurrence of a Th2 immune response [26], which accounts for decreased resistance to the infection found in these mice.

The resistance to *P. brasiliensis* infection is usually attributed to high IFN-γ, which activates macrophages and enables them to kill the fungus [5], [27], [28]. We did not detect a significant difference between the IFN-γ levels in the organs of infected gal3^+/+^ and gal3^−/−^ mice. Despite the similar IFN-γ levels, less NO was detected in the lungs of gal3^−/−^ infected mice, and produced by peritoneal macrophages obtained from them, when stimulated with *P. brasiliensis* yeast, antigens, or LPS. No difference was seen in the arginase I levels in the lungs of infected mice from both groups. Therefore, the lower NO production by gal3^−/−^ mice is probably attributed to the higher levels of IL-4 exhibited by these mice, which is known to suppress NO production by repressing IFN-γ-induced gene transcription [29], besides acting as an inducer of naive T cell differentiation into Th2 lymphocytes [30].

These two mentioned processes resulting from IL-4 overproduction can render mice more susceptible to intracellular pathogens. Indeed, IL-4 neutralization leads to protection against several fungi [31]–[33], including *P. brasiliensis* [23], [34], [35]. In *P. brasiliensis*-infected C57BL/6 mice, IL-4 neutralization leads to less severe pulmonary disease, low production of Th2 cytokines, high TNF-α and IL-12 synthesis, and low IgG1 antibodies levels [23]. The conclusion that the higher IL-4 production in gal3^−/−^ mice accounts for deviation to a Th2 immune response is strengthened by the detection in the spleen and lung of these mice of higher levels of GATA-3 mRNA, a transcriptional factor selectively expressed in Th2 lymphocytes that inhibits Th1 differentiation [36]. While Th2 lymphocytes alone may account for IL-4 overproducion; other cells, like eosinophils, may be also implicated in such a response of gal3^−/−^ mice. The involvement of mast cells is less probable because, when derived from gal3^−/−^ mice, they respond to stimuli with lower IL-4 secretion [37].

The distinct behavior of gal3^−/−^ mice toward *P. brasiliensis* infection discloses a novel way through which galectin-3 may regulate host immunity. The *R. equi* model of infection has revealed galectin-3's ability to exert a regulatory role in innate immunity by diminishing macrophage IL-1β production. Macrophages from gal3^−/−^ *R. equi*-infected mice upregulate proinflammatory and TLR2 genes, which results in enhanced cell activation and in increased resistance to the bacteria [13]. Consistent with this report, we have observed that gal3^−/−^ peritoneal macrophages develop higher TLR2 mRNA expression, in comparison with the wild type macrophages, when merely elicited with thioglycollate broth. Following stimulation with *P. brasiliensis* antigens, the TLR2 expression was remarkably greater in gal3^−/−^ macrophages, compared to that observed in other experimental conditions. Under stimulation with fungal antigen or the intact yeast, higher levels of the deactivating cytokine IL-10 were produced by gal3^−/−^ macrophages. In many aspects our results are supported by the recent work of Ferreira et al. [38] that shows that susceptible mice infected with *P. brasiliensis* develop regulatory DCs, which display increased TLR2 gene expression and IL-10 production in the presence of the yeast. Our gal3^−/−^ macrophages behave similarly in the presence of the fungal antigens, and we hypothesize that the increased TLR2 expression is implicated in the higher IL-10 production and the generation of an ineffective immune response against *P. brasiliensis*. Studies with *Candida albicans* have shown that TLR2^−/−^ mice are more resistant to this infection, a fact associated with the decreased release of anti-inflammatory cytokines, such as IL-10 [39]. Therefore, the low inflammatory response observed in gal3^−/−^ mice may be derived from the enhanced TLR2 expression and elevated IL-10 production in macrophages stimulated by the fungal antigens.

**Figure 8:**
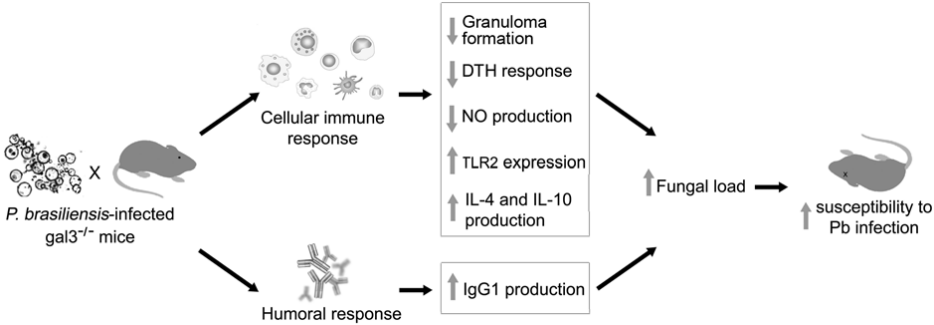
Possible role of galectin-3 during a fungal infection, as suggested by the immune response of gal3^−/−^ mice to the inoculation of *P. brasiliensis* yeasts. Mice deficient for the galectin-3 gene were infected, and several aspects of the immune response mounted by these mice are summarized in the figure.

The present study is the first demonstration that galectin-3 deficiency may polarize the immune response toward a Th2 pattern. A critical role of galectin-3 on Th1/Th2 balance has been previously suggested by us [11], [13] and others [12], [14]. The *T. gondii* model has shown that galectin-3 leads to reduced organ inflammation and Th1-polarized immune response, which does not alter mice survival to the infection [11]. During schistosomiasis, gal3^−/−^ mice present a biased Th1 cellular and humoral immune response [12]. By using a murine model of asthma, the occurrence of a lower Th2 response in gal3^−/−^ mice, compared with gal3^+/+^ mice, has been reported [14]. Our work adds new data; corroborating the idea that galectin-3 is a regulator of Th1/Th2 immunity. Because gal3^−/−^ mice, during *P. brasiliensis* infection, develop a Th2-polarized immune response and succumbed to infection, we conclude that galectin-3 is central to maintaining the intermediate resistance of C57BL/6 mice to *P. brasiliensis*, and the deletion of the galectin-3 gene renders mice susceptible to the fungal infection. Such susceptibility is associated with an unequivocal deviation toward a Th2-immune response, possibly triggered by macrophages over-expressing TLR2 and producing high IL-10 levels. Because the LPS stimulus (TLR4 agonist) induces IL-10 production that is not controlled by galectin-3, we suppose that our observations may be peculiar to fungal infections. A global idea of how susceptibility to *P. brasiliensis* infection can be influenced by galectin-3 actions on the host cellular and humoral response is given in figure 8. The results presented here, together with previous observations on the role exerted by galectin-3 in several infections, allow us to postulate that regulation of the Th1/Th2 balance by galectin-3 changes with the diversity of the initial responses triggered by different pathogens.
